# Intensity Modulation Effects on Ultrafast Laser Ablation Efficiency and Defect Formation in Fused Silica

**DOI:** 10.3390/nano15050377

**Published:** 2025-02-28

**Authors:** Dai Yoshitomi, Hideyuki Takada, Shinichi Kinugasa, Hiroshi Ogawa, Yohei Kobayashi, Aiko Narazaki

**Affiliations:** 1 National Institute of Advanced Industrial Science and Technology (AIST), 1-1-1 Umezono, Tsukuba 305-8568, Japan; h.takada@aist.go.jp (H.T.); s.kinugasa@aist.go.jp (S.K.); ogawa.h@aist.go.jp (H.O.); narazaki-aiko@aist.go.jp (A.N.); 2 The Institute for Solid State Physics, The University of Tokyo, 5-1-5 Kashiwanoha, Kashiwa 277-8581, Japan; yohei@issp.u-tokyo.ac.jp

**Keywords:** ultrafast laser processing, laser ablation, intensity modulation, femtosecond lasers, fused silica, dielectrics, defects

## Abstract

Ultrafast laser processing is a critical technology for micro- and nano-fabrication due to its ability to minimize heat-affected zones. The effects of intensity variation on the ultrafast laser ablation of fused silica were investigated to gain fundamental insights into the dynamic modulation of pulse intensity. This study revealed significant enhancement in ablation efficiency for downward ramp intensity modulation compared to the upward ramp. This effect was independent of the repetition rate ranging from 100 Hz to 1 MHz, which suggested that it originates from persistent residual effects of preceding pulses. Photoluminescence experiments indicated that the observed effect is primarily attributed to the dynamic reduction in the ablation threshold caused by the formation of defects such as non-bridging oxygen hole centers. The correlation between the sequence of intensity-modulated pulses and defect formation has been clarified. The knowledge of these correlations, combined with machine learning-based optimization methods, is useful for the optimization of the throughput and quality of ultrafast laser processing.

## 1. Introduction

Ultrafast laser processing is a pivotal technology for precise micro- and nano-fabrication, because it reduces heat diffusion from deposited energy and minimizes heat-affected zones [1,2,3]. Micro- and nano-fabrication processes, such as microhole drilling and surface nanostructuring of transparent dielectrics, are critically important for applications such as consumer electronics, flat-panel displays, optoelectronics, and medical devices. The ablation dynamics of dielectrics have been extensively studied, revealing that multiphoton excitation of valence electrons and subsequent generation of electrons through avalanche ionization are involved in the ablation process [4,5,6,7,8,9,10,11]. When multiple laser pulses irradiate a material, the preceding pulses influence the ablation caused by subsequent pulses. A reduction in the ablation threshold for multiple laser shots relative to that for a single shot, known as the incubation effect, was also addressed [7,8,9,11]. Taking such effects into account, it is crucial to dynamically adjust process parameters based on process conditions that are influenced by preceding pulses. Our future goal is to develop a data-driven ultrafast laser processing system through the integration of real-time process monitoring, AI-assisted optimization, and dynamic parameter modulation. We have demonstrated a remarkable reduction in the defect area of laser-induced periodic surface structures (LIPSSs) on a glass sample using active feedback control of laser intensity combined with real-time monitoring of surface conditions [12]. In the present study, we investigated the effects of pulse intensity modulation on the ablation of fused silica to gain fundamental insights into its impact.

Nakamura et al. employed sinusoidal intensity modulation to stabilize plasma movement for stress relaxation during the picosecond laser modification process for alkali silicate glass [13]. Hattori et al. demonstrated crack-free femtosecond laser percussion drilling of SiC through the effective suppression of shockwaves using stepwise intensity modulation [14]. Balage et al. performed a comparison of ramp modifications in terms of the ablation rate and quality with gigahertz-burst mode percussion drilling of soda-lime glass [15]. Shimahara et al. employed deep neural networks to simulate the pulse-to-pulse evolution of drilled holes in glass via ultrashort pulse laser ablation and optimized intensity modulation shapes to improve drilling efficiency [16]. As demonstrated in previous studies, despite the exploration of intensity modulation effects, the influence on ablation efficiency and the underlying physical mechanisms have yet to be fully investigated.

In this work, we investigated the effects of intensity ramp modulation on the ablation efficiency of fused silica and its underlying physical mechanisms at pulse repetition rates up to 1 MHz, which are particularly relevant for real-time process monitoring. We observed a significant enhancement in ablation efficiency under the downward ramp compared to the upward ramp. The shot-to-shot evolution analysis indicated that, in the case of a downward ramp, the initial intense pulses reduce the ablation threshold, leading to an increase in the total ablated volume of the material. From the repetition rate dependence ranging from 100 Hz to 1 MHz, the dominant residual effects of preceding pulses were found to be persistent, rather than transient phenomena such as heat accumulation or plume shielding. Finally, photoluminescence (PL) microscopy measurements revealed that more defects are generated under the downward ramp due to the intense pulses in the initial stage, predominantly contributing to the reduction in the ablation threshold through increased absorption.

## 2. Materials and Methods

**Figure 1:**
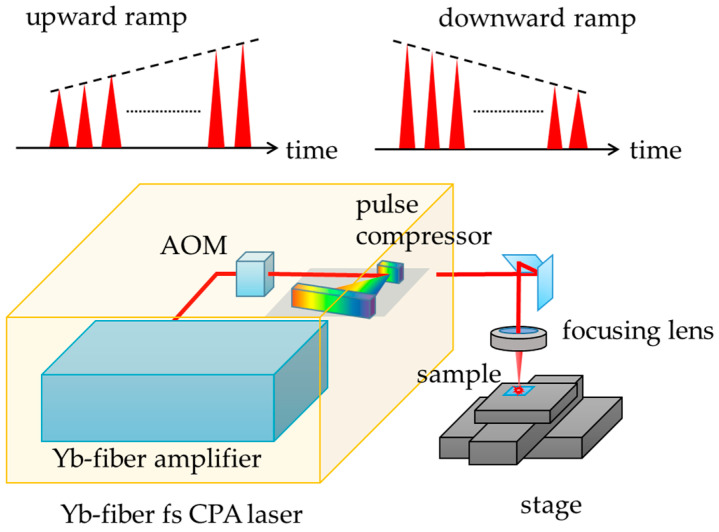
Experimental setup.

Figure 1 shows the experimental setup. A homemade automatic parameter-variable ultrashort pulse laser processing system was used as a laser source. The energy, duration, repetition rate of the pulse train and number of irradiation pulses delivered from the laser system are variable through a custom-developed program [17,18,19]. The laser is a Yb-doped fiber chirped-pulse amplification (CPA) system consisting of an oscillator, a pulse stretcher, three cascaded preamplifiers, two cascaded rod-fiber amplifiers, and a pulse compressor. It emits pulses at a wavelength of 1033 nm with an energy of up to 100 µJ at a repetition rate of 1 MHz. The pulse duration can be varied from 400 fs to 400 ps by adjusting the distance between the two gratings in the pulse compressor [17].

A double-side polished fused silica sample (30 × 30 × 1 mm^3^) was placed on a three-axis, remote-controlled stage. A linearly polarized beam with a Gaussian transverse profile was focused onto the sample using a lens with a 100 mm focal length. The spot diameter, defined at 1/*e*^2^ of the peak intensity, was determined to be 36 μm using Liu’s plot [20,21] and was in agreement with the measurement of the attenuated image acquired with a CMOS camera. The average fluence at the laser focus was estimated by dividing the pulse energy by the area of the measured spot diameter. The pulse duration was fixed at 400 fs for this experiment. The repetition rate was varied from 100 Hz to 1 MHz.

**Figure 2:**
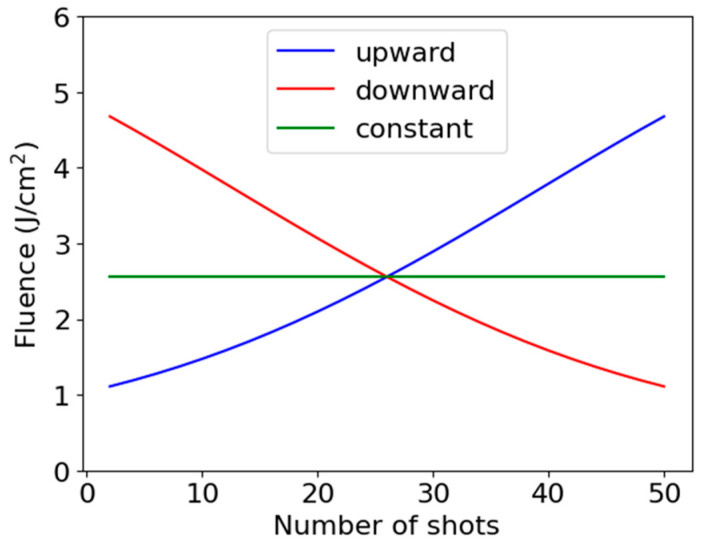
Variation in the laser fluence as a function of the number of shots under upward and downward ramps and constant fluence.

The intensity of each pulse in a 1 MHz repetitive pulse train can be controlled by using an acousto-optic modulator (AOM) placed before the pulse compressor. The AOM was also used to vary the repetition rate and select the specified number of pulses for irradiating the sample. Although an arbitrary modulation shape can be set via a computer program through a function generator, ramp intensity modulations were employed to gain fundamental insight into the physical mechanisms that underlie the modulation effects. Figure 2 illustrates the variation in the laser fluence under upward and downward ramp modulations, as well as under constant fluence (i.e., no modulation). The maximum and minimum pulse energies were set to 48 µJ and 12 µJ, corresponding to average fluences of 4.7 J/cm^2^ and 1.2 J/cm^2^, respectively. The upward and downward ramp shapes were designed to be time-reversals of each other. The total deposited energy for all shots was kept nearly identical across all modulation shapes. Although the input RF ramp signal was linear in time, the fluence variation slightly deviated from linearity due to the nonlinear response function of the AOM, where the deviation in total energy remained below 5%.

The three-dimensional profiles of the ablation craters were measured using a confocal laser scanning microscope (OLS4100, Olympus, Hachioji, Japan). The ablation depth was determined as the average depth within a 10 μm diameter circle centered on the crater’s center of gravity. The ablation volume was calculated by integrating the depth over the entire area of the crater. The morphology of the crater surface was observed with a scanning electron microscope (SEM, TM4000 plus II, Hitachi High-Tech, Tokyo, Japan). In addition, photoluminescence (PL) microscopy (NRS-5500, JASCO, Hachioji, Japan) was conducted to evaluate the number of defects induced by laser irradiation. The pump wavelength was 532 nm, and PL spectra were obtained in the wavelength range of 590 to 710 nm.

## 3. Results and Discussion

### 3.1. Ablation Efficiencies Under Upward and Downward Ramps

**Figure 3:**
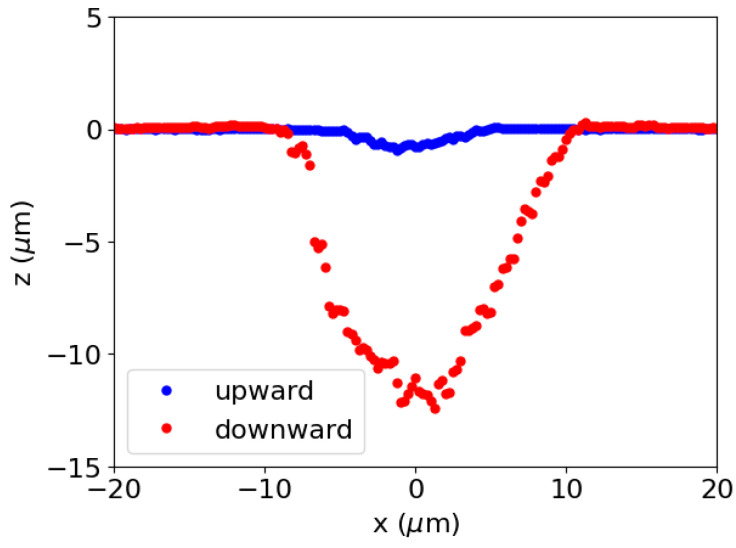
Cross-sectional profiles of ablation craters for upward and downward ramp modulations.

To investigate the effects of ramp modulation on ablation efficiency, the depths and volumes of ablation craters were compared under upward and downward ramps, as well as those under a constant fluence. The repetition rate and the number of shots were fixed at 1 MHz and 50, respectively, for this experiment. For each modulation shape, 20 craters formed under the same irradiation conditions were examined to assess statistical differences. To exclude the effects of temporal variation due to environmental instability, irradiation was performed once for each modulation shape, and the process was then repeated for 20 sets. Figure 3 shows a comparison of the cross-sectional profiles of the ablation craters under upward and downward ramps, whereas no ablation was observed under a constant fluence. The ablation depth under the downward ramp was significantly larger than that under the upward ramp.

**Figure 4:**
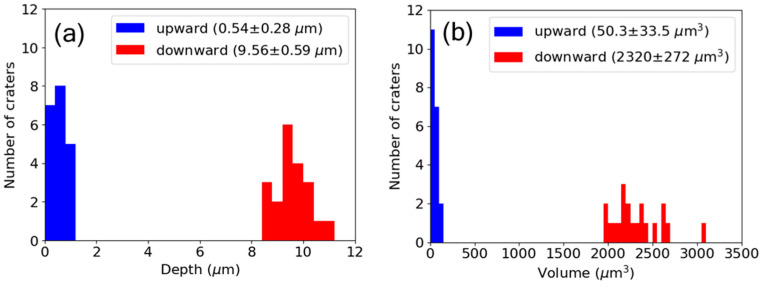
Statistical distribution of ablation (**a**) depth and (**b**) volume for upward and downward ramps.

**Figure 5:**
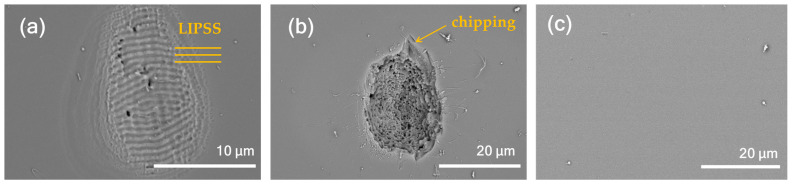
SEM images of ablation craters for (**a**) upward and (**b**) downward ramps and (**c**) constant fluence.

Figure 4 shows the statistical distributions of ablation depths and volumes for 20 craters each under upward and downward ramps. The mean values and standard deviations are shown in the inset of Figure 4. A significant difference in the mean values of the depths and volumes between the two ramps is evident. Figure 5 shows SEM images of the ablation craters for both the upward and downward ramps and constant fluence. An LIPSS that is characteristic of non-thermal ultrashort pulse laser exposure is clearly visible on the crater surface under the upward ramp shown in Figure 5a. The structure has a period of approximately 700 nm, which is close to λ/*n*, where λ = 1033 nm is the laser wavelength and *n* = 1.45 is the refractive index of fused silica. The orientation of the structure is parallel to the laser polarization. These features are consistent with the low-spatial-frequency LIPSS reported in previous studies [22,23,24]. A relatively larger volume was ablated by laser irradiation under the downward ramp, as shown in Figure 5b, which resulted in the removal of the LIPSS from the surface. The chipping observed around the ablation crater leads to the relatively larger statistical variation in the measured volumes shown in Figure 4b. In addition, no rim formation was observed, which is characteristic of non-thermal ultrafast laser ablation. For the constant fluence, no ablation was observed, as shown in Figure 5c.

### 3.2. Shot-To-Shot Ablation Dynamics

**Figure 6:**
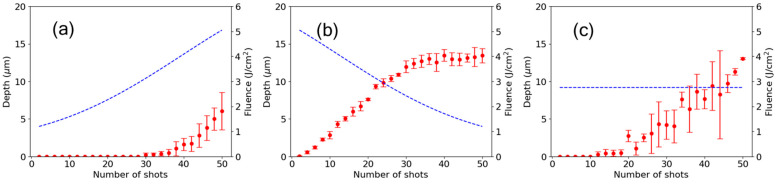
Shot-to-shot evolution of ablation depth (left axis) for (**a**) upward and (**b**) downward ramps and (**c**) constant fluence. The error bars represent the standard deviation, and the dotted curves indicate the fluence (right axis).

Shot-to-shot dynamics of ablation were experimentally studied to further investigate the cause of the significant difference in the ablation efficiency between the ramp directions. The number of shots varied from 2 to 50 in steps of 2 for all modulation shapes, while maintaining the same fluence change rate. In this experiment, the fluence was slightly increased from the range of 1.2 to 4.7 J/cm^2^ used in the experiment in Section 3.1 (Figure 2) to a range of 1.3 to 5.0 J/cm^2^. As a result, ablation occurred under constant fluence. Figure 6 shows the shot-to-shot evolution of the ablation depths for (a) upward and (b) downward ramps and (c) constant fluence. The error bars represent the standard deviation, and the dotted curves indicate the fluence. In the case of the upward ramp shown in Figure 6a, ablation did not occur until approximately 30 shots, because the initial fluence was below the ablation threshold. The fluence at which ablation began is regarded as the ablation threshold for the unablated surface of the material and was determined to be $F_{th}^{\left(up\right)}$ = 3.1 J/cm^2^. In contrast, for the downward ramp shown in Figure 6b, ablation began from the initial shot due to the fluence being higher than the single-shot ablation threshold (3.9 J/cm^2^) and ceased after approximately 36 shots. The fluence at which ablation ceased is regarded as the ablation threshold for a surface damaged by initial high-fluence pulses and was determined to be $F_{th}^{\left(down\right)}$ = 2.0 J/cm^2^. When comparing the two threshold fluences, the threshold at which ablation began for the upward ramp, $F_{th}^{\left(up\right)}$, was considerably higher than the threshold at which ablation ceased for the downward ramp $F_{th}^{\left(down\right)}$, i.e., $F_{th}^{\left(up\right)}>F_{th}^{\left(down\right)}$. This relation clearly shows that the residual effects of the preceding high-fluence pulses lead to a reduction in the ablation threshold for the downward ramp. The reduction causes the difference in the ablation depth at the final shot between the two ramps. In the constant fluence case shown in Figure 6c, the standard deviation of the ablation depth was too large to discern a clear trend. The large fluctuation is due to the given fluence being between the single-shot ablation threshold (3.9 J/cm^2^) and the multiple-shot ablation threshold (down to 2 J/cm^2^). In this fluence range, the number of shots required to initiate ablation varies depending on the surface condition.

### 3.3. Repetition Rate Dependence

**Figure 7:**
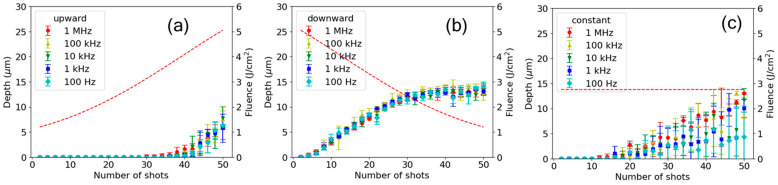
Comparison of shot-to-shot evolution of ablation depth (left axis) for (**a**) upward and (**b**) downward ramps and (**c**) constant fluence at repetition rates from 100 Hz to 1 MHz. The error bars represent the standard deviation, and the dotted curves indicate the fluence (right axis).

To investigate the timescale of the residual effects of the preceding pulses, the repetition rate dependence of the shot-to-shot evolution was examined. The experiment described in Section 3.2 was conducted at lower repetition rates down to 100 Hz. Figure 7 shows a comparison of the shot-to-shot evolution of ablation at repetition rates in the range from 100 Hz to 1 MHz for both the upward and downward ramps. The standard deviation of the depths, shown as error bars, was approximately 1 µm on average and exhibited little dependence on the repetition rates for the two ramps, as shown in Figure 7a,b. In contrast, the depth showed a large standard deviation up to 6 µm in the case of constant fluence, as shown in Figure 7c.

For the upward and downward ramps, the shot-to-shot evolution of depth showed little dependence on the repetition rate. This result suggests that transient residual effects from preceding pulses on a timescale shorter than 10 ms do not contribute to the dependence on ramp directions. We discuss the timescale of possible transient residual effects in the next section.

### 3.4. Transient Residual Effects

**Figure 8:**
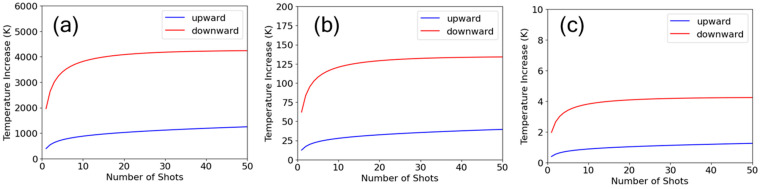
Simulation of temperature increase due to heat accumulation at the crater center for upward and downward ramps at repetition rates of (**a**) 10 kHz, (**b**) 1 kHz, and (**c**) 100 Hz. Full absorption of the incident energy was assumed for upper limit estimation.

Transient residual effects from preceding pulses may include heat accumulation, ablation plume shielding, and photoexcitation of self-trapped excitons (STEs). The temperature at the irradiated area increases due to heat accumulation when the next pulse is delivered before the heat from the previous pulse dissipates through diffusion [25,26,27]. The temperature increase at the crater center due to heat accumulation was simulated using Weber’s three-dimensional model [25]. The formula of temperature increase, Δ*T*, shown as Equation (10) in Reference [25], was modified to account for the modulated pulse energy:

(1) $$\Delta T=\frac{2}{C{\left(4\pi \kappa T_{rep}\right)}^{3/2}}\sum_{n=1}^{N}\frac{E_{n}}{n^{3/2}}\;,$$

where *C* is the volumetric heat capacity, *κ* is the thermal diffusivity, *T*_rep_ is the interval of the repetitive pulses, *N* is the number of shots, and *E_n_* is the energy of the *n*-th shot. The values of *C* = 1.6 × 10^6^ J/m^3^K and *κ* = 8 × 10^−7^ m^2^/s for fused silica were used. Since it was difficult to estimate the absorbed energy in the material, full absorption of the incident energy was assumed. The energy loss due to material removal by ablation was not considered. These assumptions provide an upper estimate of the temperature increase. Figure 8 shows the results for upward and downward ramps at repetition rates of 10 kHz, 1 kHz, and 100 Hz. The temperature increase is significantly higher under the downward ramp than under the upward ramp due to heat accumulation from the initial high-energy pulses. At the same time, the temperature increase exhibits a strong dependence on the repetition rate. At 100 Hz, the temperature increase is reduced to as small as 4 K, even in the upper estimate, which will make little contribution to ablation efficiency. Considering the experimental results showing little dependence on the repetition rate, it can be concluded that the overall influence of heat accumulation on the effects of ramp direction is minimal. Further investigation is needed to understand the reason why heat accumulation does not affect ablation efficiency even at higher repetition rates.

The shielding by the ablation plume generated by the previous pulse prevents the penetration of the following laser pulses [27,28,29]. This effect typically dissipates on a microsecond timescale, as the plume expands at speeds of kilometers per second [28,30]. Thus, the plume shielding has little influence at repetition rates below 1 kHz.

Finally, the photoexcitation of transient STEs has an effect that increases the absorption of subsequent pulses [9,10]. However, the contribution of STEs was not detected under the repetition rates below 1 MHz in this study, because their lifetime is estimated to be several hundreds of picoseconds [31,32]. A portion of the STEs relax into permanent defects, which will be discussed in Section 3.5.

### 3.5. Photoluminescence Microscopy to Evaluate Defect Formation

**Figure 9:**
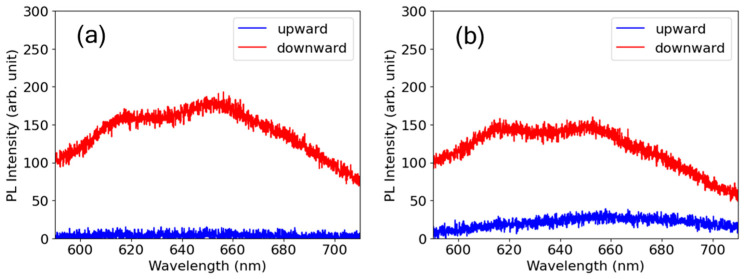
Comparison of PL spectra from ablation craters under upward and downward ramps, both with identical accumulated fluence, *F*_acc_. (**a**) *F*_acc_ = 75 J/cm^2^ with 34 shots for the upward ramp and 18 shots for the downward ramp. (**b**) *F*_acc_ = 100 J/cm^2^ with 40 shots for the upward ramp and 26 shots for the downward ramp.

A PL microscopy experiment was conducted to evaluate the defect formation induced by laser irradiation. A broad PL spectrum with two peaks at 620 and 650 nm was observed upon pumping with a wavelength of 532 nm. The spectrum originates from the non-bridging oxygen hole center (NBOHC), which is a dangling bond of oxygen with an unpaired electron [33,34,35,36]. In addition to the NBOHCs, other defects, such as oxygen deficiencies, may have formed and could be observed when pumping was performed at shorter wavelengths. Figure 9 shows a comparison of the PL spectra from NBOHC defects on the ablation craters for both upward and downward ramps. The spectra for the two ramps were compared for craters with irradiation of equal accumulated fluences of *F*_acc_ = 75 J/cm^2^ in (a) and *F*_acc_ = 100 J/cm^2^ in (b). The PL intensity is notably higher for the downward ramp than for the upward ramp at both values of *F*_acc_, which indicates that more defects were created despite the smaller number of shots for the downward ramp than for the upward ramp. Intense pulses at the initial stages of the downward ramp led to the formation of more defects due to the nonlinear nature of multiphoton absorption. An increase in the number of defects enhances absorption, which leads to a reduction in the ablation threshold. Apart from intensity modulation, the reduction in the ablation threshold for multiple-shot irradiation compared to that for single-shot irradiation, which is recognized as incubation, has been discussed in prior works and is related to defect formation using a simple rate equation model [7,8]. It can be inferred that similar effects arising from defect formation occur, leading to changes in ablation efficiency during intensity-modulated ablation.

## 4. Conclusions

This work thoroughly investigated the effects of laser pulse intensity modulation on the ultrafast ablation efficiency of fused silica at repetition rates below 1 MHz with consideration of its potential for future advancements in the active optimization and real-time monitoring of ultrafast laser processing. A significant enhancement in ablation efficiency was observed for the downward ramp modulation compared to that of the upward ramp. The enhanced ablation efficiency under the downward ramp is attributed to a reduction in the ablation threshold, caused by increased defect formation due to intense pulse exposure during the initial stage, as directly confirmed by PL microscopy measurements. The transient residual effects of preceding pulses do not play a significant role in the enhancement of ablation efficiency. A correlation between the sequence of intensity-modulated pulses and defect formation has been clarified. Considering that defects are relevant to processing quality, knowledge of such correlation combined with machine learning-based optimization is considered to be useful for optimization of the throughput and quality of ultrashort pulse laser processing.

## Data Availability

The data that support the findings of this study are available from the corresponding author upon reasonable request.

## Abbreviations

The following abbreviations are used in this manuscript:

LIPSS	Laser-Induced Periodic Surface Structure
CPA	Chirped-Pulse Amplification
AOM	Acousto-Optical Modulator
SEM	Scanning Electron Microscope
PL	Photoluminescence
STE	Self-Trapped Exciton
NBOHC	Non-Bridging Oxygen Hole Center
